# Complications and failure after Kock continent ileostomy: A systematic review and meta-analysis

**DOI:** 10.1007/s10151-024-03018-x

**Published:** 2024-10-01

**Authors:** S. H. Emile, Z. Garoufalia, S. Mavrantonis, P. Rogers, S. H. Barsom, N. Horesh, R. Gefen, S. D. Wexner

**Affiliations:** 1 Ellen Leifer Shulman and Steven Shulman Digestive Disease Center, Cleveland Clinic Florida, 2950 Cleveland Clinic Blvd, Weston, FL 33331 USA; 2 General Surgery Department, Colorectal Surgery Unit, Mansoura University Hospitals, Mansoura, Egypt; 3 Bart’s and the London School of Medicine and Dentistry, London, UK; 4 Internal Medicine Department, University of South Dakota, Sanford School of Medicine, Sioux Falls, South Dakota USA; 5 Department of Surgery and Transplantation, Sheba Medical Center, Ramat-Gan, Israel; 6 Department of General Surgery, Hadassah Medical Organization and Faculty of Medicine, Hebrew University of Jerusalem, Jerusalem, Israel

**Keywords:** Kock pouch, Continent ileostomy, Complications, Failure, Systematic review, Meta-analysis

## Abstract

**Background:**

A significant number of patients experience complications of the Kock pouch (KP) warranting revision or excision. This systematic review aimed to assess the pooled prevalence and risk factors for complications and failure of the KP.

**Methods:**

This Preferred Reporting Items for Systematic Reviews and Meta-analyses (PRISMA)-compliant systematic review (CRD42023416961) searched PubMed, Scopus, and Web of Science for studies on adult patients with Kock continent ileostomy published after the year 2000. The main outcome measures were revision, complications, and failure of the KP. Risk factors for complications and failure were assessed using a meta-regression analysis. Risk of bias was assessed using the ROBINS-1 tool. A proportional meta-analysis of the main outcomes was performed.

**Results:**

A total of 19 studies (2042 patients) were included. The weighted mean prevalence of complications was 60.4% [95% confidence interval (CI): 46.1–74.7%], of pouch revision was 46.6% (95% CI: 38.5–54.7%), and of pouch failure was 12.9% (95% CI: 9.3–16.4%). Studies conducted in the USA had a mean failure prevalence of 12.6% (95% CI: 6.2–18.9%) comparable to studies conducted in Europe (11.1%; 95% CI: 7.5–14.7%). Factors associated with higher complications were increased body mass index (BMI) and previous ileoanal pouch anastomosis (IPAA); however, these factors were not associated with increased pouch failure.

**Conclusions:**

The KP is a highly complex operation as shown by a pooled complication prevalence of 60%, and thus, it should be only performed by experienced surgeons. Despite the high prevalence of complications and need for revisional surgery, patients are keen to preserve their KP. Increased BMI and a previous failed IPAA are risk factors for pouch complications, but not failure.

**Supplementary Information:**

The online version contains supplementary material available at 10.1007/s10151-024-03018-x.

## Introduction

Kock continent ileostomy, also known as the Kock pouch (KP), was introduced in 1969 by Professor Nils Kock from Gothenburg, Sweden [1]. The concept of the procedure was to provide permanent ileostomies with continence by the creation of an inward-facing nipple valve [2]. The KP was mainly used in patients with mucosal ulcerative colitis (MUC) and familial adenomatous polyposis (FAP) as an alternative to a Brooke end ileostomy.

Despite the sound concept of the procedure, the KP was found to be associated with a considerable rate of complications that warrant at least a single revision. Perhaps the most common indication for revision of the KP is nipple-valve-related complications, especially valve slippage [3]. A systematic review [4] reported the incidence of valve slippage to range between 15.8% and 57%. Valve slippage has been attributed to the effect of bulky mesentery that prevents the attachment of the two walls of the valve, occurring most commonly at the mesenteric aspect [5].

In addition, nipple-valve-related complications include valve dysfunction, stricture, and difficult intubation. The valve-related complications of the KP stimulated the development of alternative reservoir techniques. These include the Barnett intestinal reservoir in which the mesenteric side is reinforced using an intestinal collar around the exit conduit [6], and the non-intussuscepting continent T-pouch [7]. In 1979 Sir Alan Parks and Mr. John Nicholls [8] described the S-pouch; in 1980 Utsunomiya et al. [9] described the J-pouch, which has widely replaced the KP as the preferred method to avoid a Brooke ileostomy.

A recent systematic review [3] explored the long-term outcomes of the Kock pouch. This review was undertaken in the context of continent ileostomies and therefore included the Barnett intestinal reservoir and T-pouch. Since the majority of revisions surgery and excisions of the KP are due to complications, a detailed analysis of the individual complications and their risk factors was undertaken to improve the current outcomes of the KP. Therefore, the present systematic review aimed to assess the pooled prevalence and risk factors for complications and failure of the KP as reported in all studies published after the year 2000.

## Methods

### Registration and reporting

The protocol of this systematic review was registered in the International Prospective Register of Systematic Reviews (PROSPERO; CRD42023416961). The review was reported in keeping with the guidelines of the Preferred Reporting Items for Systematic Reviews and Meta-analyses (PRISMA 2020) [10].

### Literature search

Two authors (S.E.and P.R.) independently performed a systematic literature search for complications following KP creation, querying PubMed, Scopus, and Web of Science for published and ahead-of-publication studies from January 2000 through April 2023. A snowball search strategy was employed, searching the references section of the studies initially retrieved. In addition, the PubMed function “related articles” was activated to look for more potentially eligible articles.

Duplicate reports and conference abstracts without access to the full text were excluded, and the remaining studies were stepwise screened by title and abstract first, and then the full-text versions of selected articles were independently reviewed by one of two authors (S.E. or S.B.) to assess for eligibility. The process of article selection and screening was supervised by the senior author (S.D.W.), who has extensive experience with creating and revising Kock pouches.

We used the following keywords in the search process “Kock pouch,” “K pouch,” “Continent ileostomy,” “complications,” “outcome,” “revision,” “excision,” and “failure.” In addition, we used the following medical subject headings (MeSH) terms: (ileostomy), (pouch), (complications), and (outcome). The following syntax combination was used for the literature search: (Kock pouch OR Continent ileostomy) AND (Complications OR Outcome OR Revision OR Excision OR Failure).

### Study selection

Both observational and randomized studies were considered eligible for inclusion. The studies included were required to have full text available in English and to fulfill the following Patients, Intervention, Comparator, and Outcome (PICO) criteria:

- P (*Patients)*: patients with inflammatory bowel disease (IBD), (FAP), colorectal cancer, and other conditions treated with total colectomy or proctocolectomy.
- I (*Intervention)*: Kock continent ileostomy.
- C (*Comparator)*: Regular Brooke ileostomy, ileal pouch anal anastomosis (IPAA), or no comparator.
- (*Outcome*): Complications, revision, excision, and failure

We excluded case reports and case series entailing less than 20 patients, animal studies, editorials, previous reviews and meta-analyses, studies that did not report the complications of KP, and studies that assessed other techniques of continent ileostomy such as T-pouch and Barnett ileostomy. In the case of overlapping studies, the most recent or larger study reporting the outcomes of this review was included.

### Assessment of study quality and risk of bias

Two authors (S.E. and Z.G.) independently assessed the risk of bias in the studies using the ROBINS-1 tool [11], and conflicts in the assessment were resolved by mutual agreement. The publication bias among the studies was visually assessed by inspection of a funnel plot of the standard error of the prevalence of total complications, revision, and failure of KP against the prevalence of these outcomes. The Egger’s regression test was also used to assess publication bias for the main outcomes. The symmetry of the funnel plot and the presence of 95% of the studies near the straight vertical line in the plot indicated the absence of publication bias. The certainty of the evidence of each outcome was graded as very low, low, moderate, and high using the Grading of Recommendations Assessment, Development, and Evaluation (GRADE) approach [12].

### Data collected

Two authors (S.E. and S.B.) extracted the following data from the studies into a preformed Excel sheet template:

- Authors, duration, country, and design of the study.
- Number, age, sex, and body mass index (BMI) of the patients
- Indications for KP.
- Previous IPAA.
- Total and individual complications.
- Pouch revision and failure.
- Follow-up duration.

### Outcome measures

The primary outcome of this review was the pooled prevalence of total and individual complications after the KP and the associated risk factors. Complications were defined as any deviation from the normal expected course after the KP. Total complications included all complications reported in the original studies. Individual complications that were reported by multiple studies were selected to be pooled.

Secondary outcomes were the pooled prevalence of pouch revision or exicsion and risk factors associated with such failures. Revision was defined as the need for reoperation to revise the pouch without the need for excising. Failure was defined as the need for pouch removal with the formation of a Brooke ileostomy either due to permanent pouch dysfunction or complications.

### Statistical analysis

A proportional meta-analysis was conducted using open-source, cross-platform software for advanced meta-analysis, openMeta [Analyst] ^™^ version 12.11.14. The weighted mean prevalence and the corresponding 95% confidence interval (CI) of complications, revision, excision, and failure of the KP were calculated using a random-effect or fixed-effect model based on the amount of statistical heterogeneity assessed by the inconsistency (*I*^2^) statistics (low if *I*^2^ < 25%, moderate if *I*^2^ = 25–75%, and high if I^2^ > 75%). The binary random-effect model was used to pool data when significant (*p* < 0.1) statistical heterogeneity was observed. Subgroup meta-analyses of the prevalence of complications according to study year, country, surgery indication, follow-up, and sample size were conducted. A random-effect meta-regression analysis was conducted to assess the factors significantly associated with the main outcomes of this review and the results were reported as slope coefficient (SE) and *p*-value. Owing to the low statistical power of meta-regression analyses, a *p*-value < 0.1 was considered statistically significant.

## Results

### Characteristics of patients and studies

**Fig. 1:**
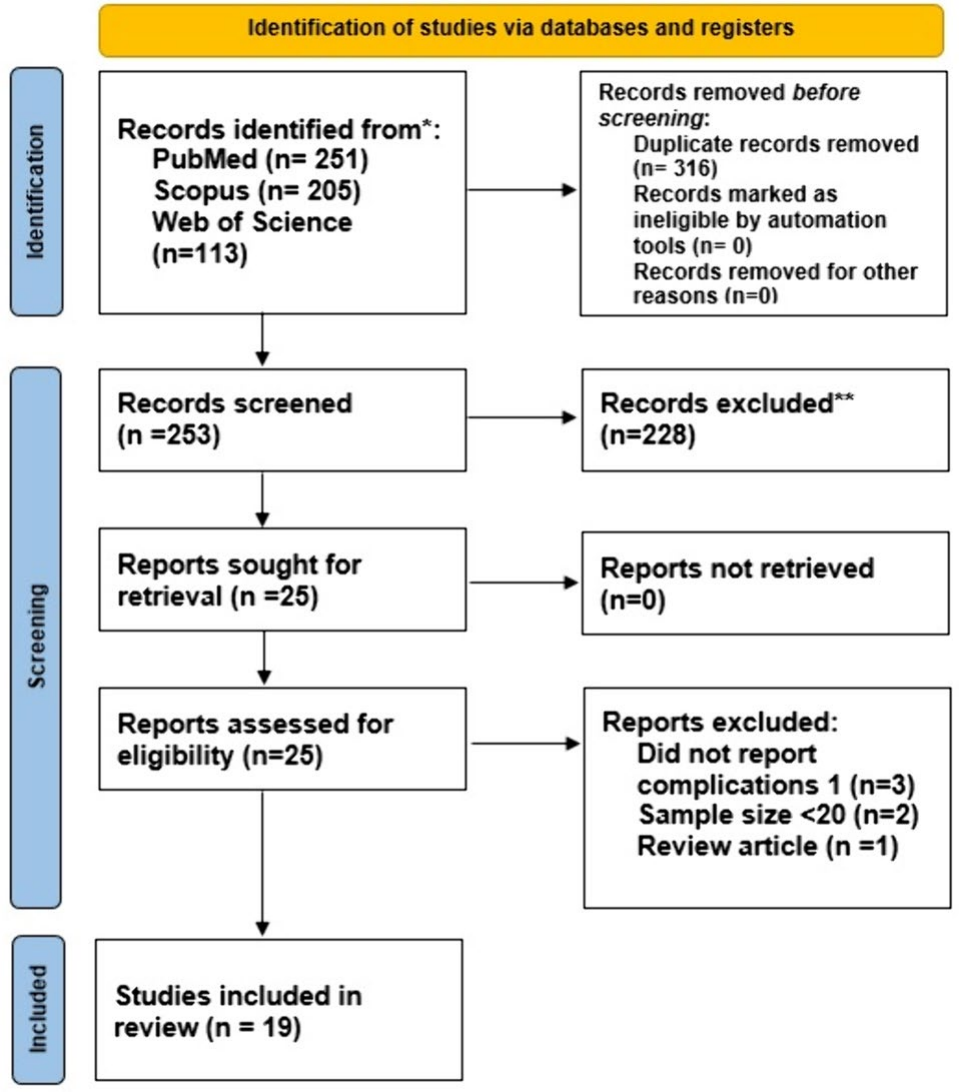
PRISMA flow chart

After the initial screening of 569 articles, 19 [13–31] were included in this systematic review (PRIMSA flow chart, Fig. 1). The studies were published between 2005 and 2022 and were based in the USA (*n* = 7), Sweden (*n* = 3), Norway (*n* = 3), Germany (*n* = 2), Finland (*n* = 1), France (*n* = 1), Italy (*n* = 1), and the Netherlands (*n* = 1). All studies were retrospective cohort studies.

Overall, 2042 patients were included in the review, with a median age of 38.2 (range, 23.1–46.4) years and a female-to-male ratio of 1.17:1. KP was performed after failed IPAA in 322 (15.8%) patients. The number of patients operated on per study year ranged from 0.96 to 22.7, with a mean of 5.2 patients per year (Table 1). In 16 studies, the indication for a KP was IBD, whereas in 10 studies it was FAP, and in seven studies, functional disorders including colonic inertia and Hirschsprung’s disease were the indications for KP (Table 2).

**Table 1:** Characteristics of patients and studies

Study	Duration	Country	Number	Number per year of study	Age in years	Male	BMI in kg/m^2^
Ecker et al., 2022	1988–2015	Germany	26	0.96	42.5	13	NA
Risto et al., 2022	1964–1996	Sweden	727	22.7	36	378	NA
Ecker et al., 2022	1986–2015	Germany	77	2.6	46.4	35	NA
Risto et al., 2021	1980–2016	Sweden	85	2.4	36	43	NA
Aytac et al, 2019	1982–2013	USA	134	4.3	37	48	23.5
Aytac et al., 2017	1978–2013	USA	48	1.4	33	14	23
Sunde et al., 2017	2000–2013	Norway	47	3.6	40	NA	NA
Mukewar et al., 2014	2002–2011	USA	36	4	23.1	17	NA
Parc et al., 2011	1973–2007	France	49	1.4	42	15	NA
Lian et al., 2009	1982–2007	USA	64	2.6	36.5	27	23.4
Hoekstra et al., 2009	1996–2007	The Netherlands	28	2.5	46	10	NA
Wasmuth et al., 2009	1983–2007	Norway	63	2.6	36.5	27	NA
Denoya et al., 2008	1998–2003	USA	31	6.2	NA	NA	NA
Wasmuth et al., 2007	1983–2002	Norway	50	2.6	37	20	NA
Nessar et al., 2006	1974–2001	USA	330	12.2	34.9	143	NA
Delaini et al., 2005	1967–1972	Italy	59	11.8	NA	NA	NA
Berndtsson et al., 2004	1967–1974	Sweden	68	9.7	30	25	NA
Castillo et al., 2005	1993–2003	USA	24	2.4	22–73	5	NA
Lepistö et al., 2005	1972–2000	Finland	96	3.4	34	56	NA

*BMI* body mass index; *NA* not available

**Table 2:** Indications for Kock pouch

Study	Indication	Type of Kock pouch	Previous IPAA
Ecker et al., 2022	Failed IPAA for IBD and FAP	Primary	26
Risto et al., 2022	Failed IPAA, IBD	Primary	22
Ecker et al., 2022	Failed IPAA, IBD, FAP, rectal cancer, slow-transit constipation	Revisional after failed Kock pouch	15
Risto et al., 2021	Failed IPAA, IBD, FAP	Primary	8
Aytac et al., 2019	Failed IPAA, IBD, FAP, motility disorders	Primary	67
Aytac et al., 2017	Failed IPAA, Crohn’s disease	Primary	4
Sunde et al., 2017	MUC	Primary	0
Mukewar et al., 2014	Failed IPAA, IBD	Primary	36
Parc et al., 2011	Failed IPAA, IBD, FAP, Hirschsprung’s disease, CRC	Primary	32
Lian et al., 2009	Failed IPAA, IBD, FAP, colonic inertia	Primary	64
Hoekstra et al., 2009	Failed IPAA, IBD, FAP, CRC, slow transit constipation	Primary	3
Wasmuth et al., 2009	Avoid ileostomy (7), remove ileostomy (23), IPAA failure (2010), ileostomy failure (22)	Primary	10
Denoya et al., 2008	NA	Revisional after failed Kock pouch	NA
Wasmuth et al., 2007	Failed IPAA, IBD, FAP	Primary	5
Nessar et al., 2006	Failed IPAA, IBD, FAP, colonic inertia, imperforate anus	Primary	20
Delaini et al., 2005	Crohn’s colitis	Primary	0
Berndtsson et al., 2004	Ulcerative colitis	Primary	0
Castillo et al., 2005	Failed IPAA, IBD, FAP, colonic inertia	Primary	7
Lepistö et al., 2005	Failed IPAA, IBD, FAP	Primary	3

*IPAA* ileal pouch anal anastomosis, *FAP* familial adenomatous polyposis, *IBD* inflammatory bowel disease, *MUC* mucosal ulcerative colitis, *CRC* colorectal cancer, *NA* not available

### Total and individual complications

**Fig. 2:**
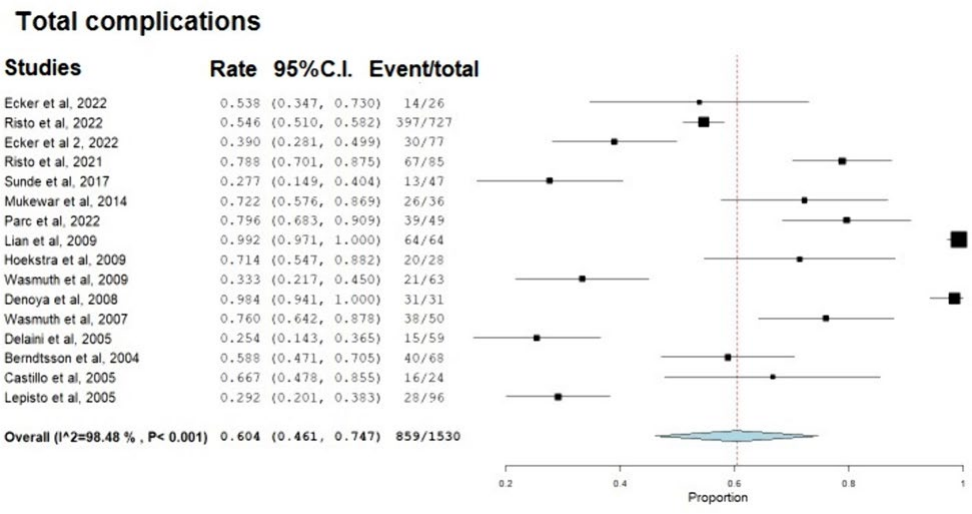
Forest plot depicting the weighted mean prevalence of total complications after Kock pouch

**Fig. 3:**
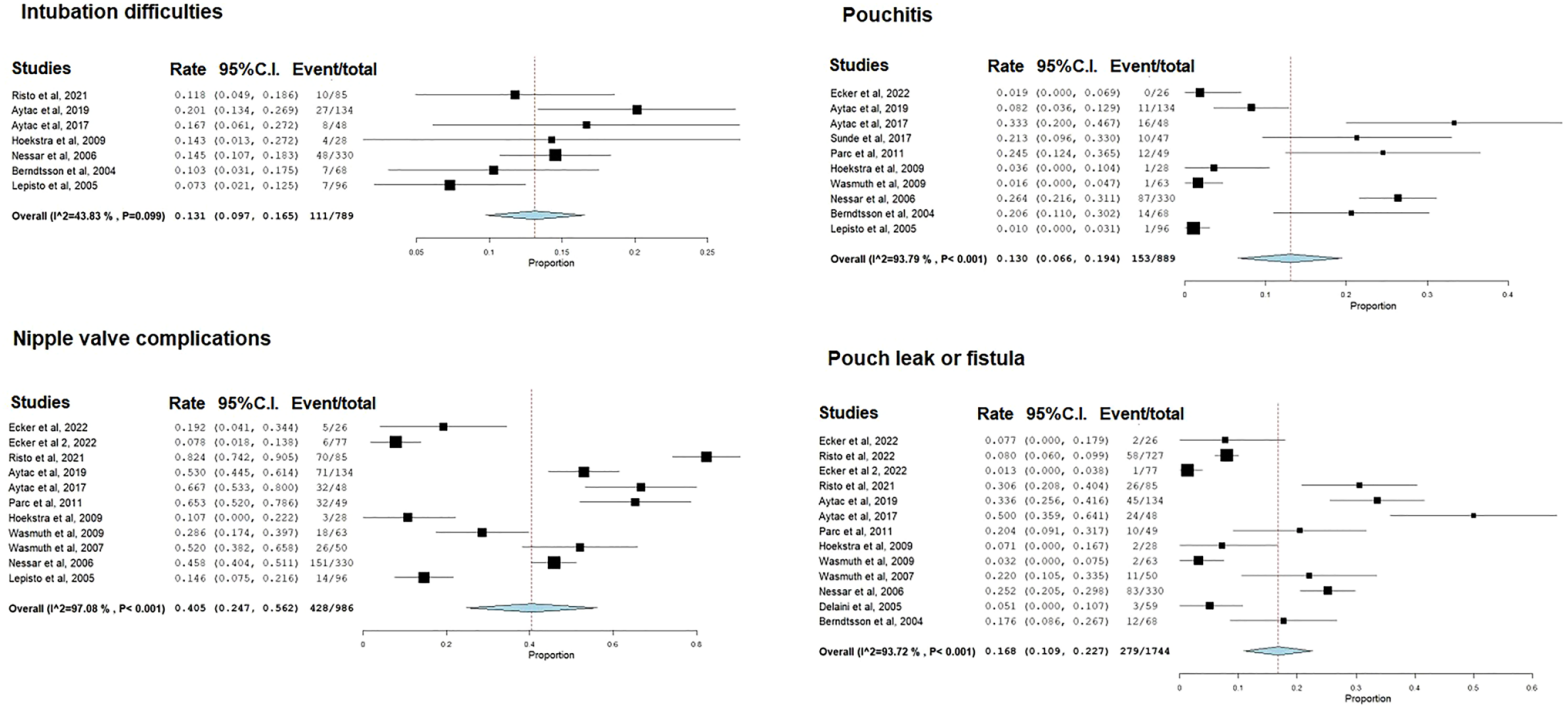
Forest plot depicting the weighted mean prevalence of nipple valve complications, difficult intubation, pouchitis, and leak or fistula after Kock pouch

The weighted mean prevalence of total complications was 60.4% (95% CI: 46.1–74.7%, *I*^2^ = 98%; Fig. 2). The weighted mean prevalence of nipple valve complications was 40.5% (95% CI: 24.7–56.2%, *I*^2^ = 97%), difficult intubation 13.1% (9.7–16.5%, *I*^2^ = 43.8%), pouch leak/fistula 16.8% (95% CI: 10.9–22.7%, *I*^2^ = 93.7%), pouchitis 13% (95% CI: 6.6–19.4%, *I*^2^ = 93.8%), abscess 11.9% (95% CI: 0.2–23.6%, *I*^2^ = 81.1%), hernia 8.6% (95% CI: 4.3–13%, *I*^2^ = 88.1%), and short-bowel syndrome 2.5% (95% CI: 0.3–4.7%, *I*^2^ = 0; Fig. 3). The total and individual complications reported in each study are summarized in Table 3.

**Table 3:** Complications after Kock pouch

Study	Number of patients	Total complications	Intubation difficulties	Valve complications	Pouch fistula/leak	Pouchitis	Hernia
Ecker et al., 2022	26	14	NA	5	2	0	0
Risto et al., 2022	727	397	NA	NA	58	NA	51
Ecker et al., 2022	77	30	NA	6	1	NA	1
Risto et al., 2021	85	67	10	70	26	NA	7
Aytac et al., 2019	134	204	27	71	45	11	18
Aytac et al., 2017	48	106	8	32	24	16	9
Sunde et al., 2017	47	13	NA	NA	NA	10	NA
Mukewar et al., 2014	36	26	NA	6	NA	8	NA
Parc et al., 2011	49	39	NA	32	10	12	NA
Lian et al., 2009	64	64	NA	22	9	8	10
Hoekstra et al., 2009	28	20	4	3	2	1	NA
Wasmuth et al., 2009	63	21	NA	18	2	1	NA
Denoya et al., 2008	31	31	NA	29	2	NA	NA
Wasmuth et al., 2007	50	38	NA	26	11	NA	NA
Nessar et al., 2006	330	512	48	151	83	87	51
Delaini et al., 2005	59	15	NA	NA	3	NA	NA
Berndtsson et al., 2004	68	40	7	NA	12	14	NA
Castillo et al., 2005	24	16	5	13	1	NA	1
Lepistö et al., 2005	96	28	7	14	6	1	NA

#### Subgroup analyses

The pooled complication prevalence in the studies published after 2010 was slightly lower than that in the studies published before 2010 [58.1% (95% CI: 45.1–71.1%) versus 59.9% (95% CI: 41–78.7%)]. Studies with follow-up > 10 years (*n* = 12) had a pooled complication prevalence of 54.6% (95% CI: 43–66.2%) compared with 71% (95% CI: 55–87%) in studies with follow-up < 10 years. Studies conducted in the USA had a mean complication prevalence of 89.9% (95% CI: 81.1–98.5%), higher than that in studies conducted in European countries [52.2% (95% CI: 41.5–62.9%)]. After exclusion of the two largest studies [14, 27], the weighted mean rate of total complications was 60.8% (95% CI: 45.9–75.7%). The pooled prevalence of complications in studies with > 5 patients per year was 59.6% (95% CI: 29.6–89.6%). Studies (*n* = 6) that mainly included patients with IBD had a pooled complications prevalence of 48.5% (95% CI: 35.4–61.6%), whereas the pooled complication prevalence in the studies that included patients with slow-transit constipation, inertia, or other motility disorders was higher, at 73.8% (9%CI: 49.1–98.5). A leave-one-out meta-analysis did not reveal a significant change in the pooled prevalence of complications on the exclusion of each study (Supplementary Fig. 1).

#### Meta-regression analysis

Factors associated with higher complications were increased BMI (SE: 0.992, *p* < 0.001), previous IPAA (SE: 0.008, *p* = 0.002), and country of the study (SE: − 0.334, *p* = 0.002). Age (SE: − 0.004, *p* = 0.656), male sex (SE: − 0.0001, *p* = 0.528), and study year (SE: − 0.043, *p* = 0.722) were not significantly associated with complications after KP creation.

### Pouch revision and failure

**Fig. 4:**
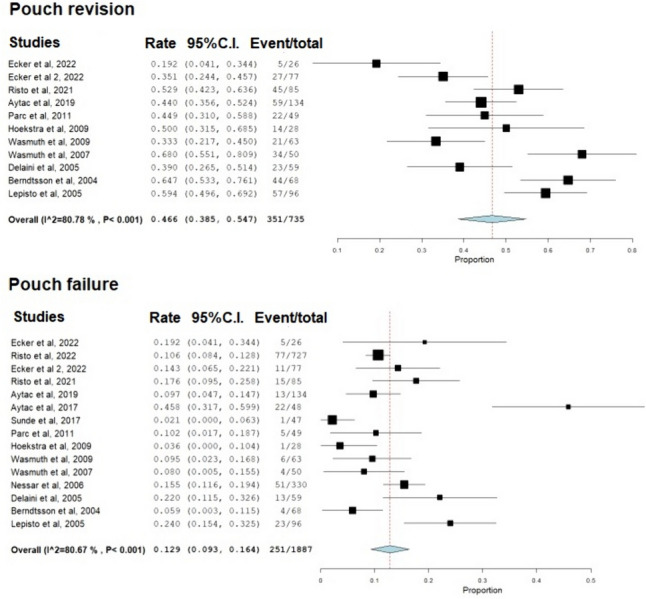
Forest plot depicting the weighted mean prevalence of Kock pouch revision and failure

The median follow-up period was 12.7 (range, 3.7–31) years. The weighted mean prevalence of pouch revision was 46.6% (95% CI: 38.5–54.7%, *I*^2^ = 80.8%), and of pouch failure was 12.9% (95% CI: 9.3–16.4%, *I*^2^ = 80.7%; Fig. 4). A summary of failure and revision prevalence in each study is shown in Table 4.

**Table 4:** Revision and failure of Kock pouch

Study	Number of patients	Revision	Failure	Follow-up in years
Ecker et al., 2022	26	5	5	7.5
Risto et al., 2022	727	1033	77	27
Ecker et al., 2022	77	27	11	30
Risto et al., 2021	85	45	15	24
Aytac et al., 2019	134	59	13	6
Aytac et al., 2017	48	53	22	19
Sunde et al., 2017	47	NA	1	3
Mukewar et al., 2014	36	NA	3	21
Parc et al., 2011	49	22	5	20.5
Lian et al., 2009	64	29	3	5
Hoekstra et al., 2009	28	14	1	3.75
Wasmuth et al., 2009	63	21	6	10.5
Denoya et al., 2008	31	12	2	7
Wasmuth et al., 2007	50	34	4	12
Nessar et al., 2006	330	NA	51	11
Delaini et al., 2005	59	23	13	27
Berndtsson et al., 2004	68	44	4	31
Castillo et al., 2005	24	14	2	5.5
Lepistö et al., 2005	96	57	23	18

#### Subgroup analyses

The weighted mean pouch failure prevalence in the studies published after 2010 (13.2%; 95% CI: 8.2–18.1%) was higher than that in the studies published before 2010 (10.4%; 95% CI: 6.2–14.6%). Studies with a follow-up of > 10 years (*n* = 12) had a mean failure rate of 14.4% (95% CI: 10.6–18.2%) compared with 5.9% (95% CI: 2.8–9%) in studies with a follow-up of < 10 years. Studies conducted in the USA had a mean failure prevalence of 12.6% (95% CI: 6.2–18.9%), comparable to studies conducted in Europe (11.1%; 95% CI: 7.5–14.7%). After exclusion of the two largest studies [14, 27], the weighted mean prevalence of pouch failure was 11.6% (95% CI: 7.8–15.4%) and of pouch revision was 46.7% (95% CI: 39.9–53.5%). The pooled prevalence of failure and revision in studies that included > 5 patients per year was 11.5% (95% CI: 7.3–15.7%) and 48% (95% CI: 29.7–66.3%), respectively. A leave-one-out meta-analysis did not reveal a significant change in the pooled prevalence of failure and revision on the exclusion of each study (Supplementary Fig. 1).

**Fig. 5:**
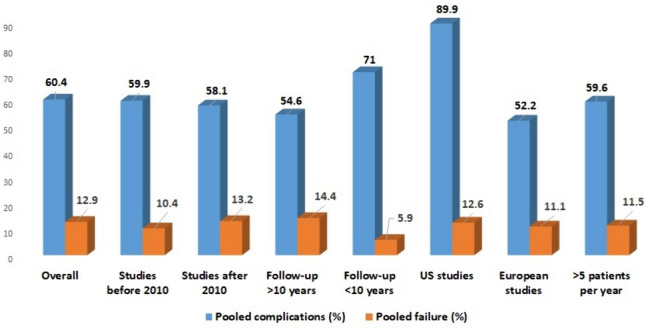
Pooled prevalence of pouch complications and failure in the sensitivity analyses

A summary of the pooled prevalence of pouch complications and failure in the sensitivity analyses is shown in Fig. 5.

#### Meta-regression analysis

None of the examined variables were associated with pouch failure [age (SE: − 0.006, *p* = 0.222), male sex (SE: − 0.0001, *p* = 0.778), previous IPAA (SE: − 0.0001, *p* = 0.710), study year (SE: 0.020, *p* = 0.567), country of the study (SE: − 0.082, *p* = 0.114), complications (SE: − 0.001, *p* = 0.971), nipple valve complications (SE: 0.001, *p* = 0.473), pouchitis (SE: − 0.006, *p* = 0.164), and pouch leak/fistula (SE: − 0.0001,  = 0.795)].

### Risk of bias and certainty of evidence

A total of 12 studies had some concern of bias, and 7 were at high risk of bias (Appendix Table 1). There was no evidence of publication bias for complications, pouch revision, and failure as shown by the insignificant *p*-values of the Egger’s regression test (*p* = 0.381, *p* = 0.571, and *p* = 0.388, respectively; Supplementary Fig. 2). Based on the GRADE assessment, the main outcomes of this review had very low certainty of evidence because of the high risk of bias, inconsistency, and imprecision as shown in Appendix Table 2.

## Discussion

The findings of the present systematic review reinforced the previous notion regarding the considerable prevalence of complications and the need for revision surgery after Kock continent ileostomy. The present study was a dedicated analysis of complications after Kock pouch creation, providing further insights into the risk factors for complications and variations in their prevalence. The selection criteria for inclusion into the present review were predefined to ensure a contemporary cohort of patients. In addition, by excluding case series involving < 20 patients, the small study effect was minimized [32]. Indications for the creation of the KP varied across the studies with the most common indication being IBD and FAP.

According to our analysis, the pooled prevalence of complications of KP was approximately 60%. Such a high rate is expected given the pouch design that entails a nipple valve predisposed to various complications. Unfortunately, the prevalence of complications did not decrease over time, as more recent studies published after the year 2010 reported a similar complication prevalence to earlier studies. Interestingly, the pooled complication prevalence in studies with follow-up longer than 10 years was lower than that of studies with shorter follow-ups. This observation, despite being counterintuitive, might be related to the type and pattern of complications or perhaps attributable to reporting bias in studies with longer follow-up durations.

The most common complications were related to the nipple valve, including valve dysfunction, slippage, stricture, and leak, accounting for a pooled prevalence of 40.5%. It is estimated that approximately every second or third patient would have a revision operation of the nipple valve within 20 years after primary continent ileostomy construction [15, 33]. Another common issue with the KP is difficult intubation, with a pooled prevalence of 13.1%. The common cause of difficult intubation of KP is partial nipple valve slippage [27]. One suggested method to avoid difficult intubation is to construct the nipple valve with an isoperistaltic intussusception [34].

Akin to the J-pouch in IPAA, the KP is susceptible to pouchitis, albeit to a much lesser degree as the pooled prevalence of pouchitis was 13.1%, compared with a cumulative incidence of 48% after the J-pouch [35, 36]. Short-bowel syndrome was reported in three studies [17, 22, 28], with a pooled prevalence of 2.5%. This complication usually occurs after the excision of the continent ileostomy. It has been suggested that having a continent ileostomy after a failed IPAA may increase the risk of failure, with subsequent pouch excision resulting in short bowel syndrome. However, the study by Aytac et al. [17] found the risk for short-bowel syndrome after a continent ileostomy is similar between patients with or without a history of failed previous IPAA.

Patients recognized to be at an increased risk of complications after the KP in our analysis were obese or had previous failed IPAA. This finding is discordant with a previous study [17] that concluded a continent ileostomy is safe in patients with a prior failed IPAA, demonstrating no increased risk of postoperative complications. Revision of IPAA and J pouch into a KP is technically demanding and requires substantial experience. If the failed J-pouch can be used as the continent ileostomy reservoir, the length of the remaining bowel can be maintained, and thus, the risk of short-bowel syndrome would be minimized in the event that further revisions are needed. However, in the classical scenario where the failed J-pouch is excised and a continent ileostomy is created as a new reservoir, up to 30% of the bowel would be compromised should the continent ileostomy need to be excised in the future [17]. Older reports concluded that conversion of the failing IPAA to a continent ileostomy, rather than to a Brooke ileostomy, is rewarding provided that the surgeon is very familiar with the technique and the primary disease is suitable for pouch surgery [37]. A more recent study [13] highlighted some strategic considerations when converting a failed IPAA to KP. Namely, the complexity of the conversion in terms of the extent of pouch reconstruction, classified as complete, partial, or none, should be considered. Complex conversion procedures may confer similar long-term outcomes to new constructions.

The high prevalence of complications of KP was paralleled with a significant prevalence of pouch revisions, most of which were to correct nipple valve issues. This high prevalence of pouch revisions may indicate that patients were keen to preserve the KP despite the need for frequent revisions instead of converting to a regular Brooke ileostomy. This observation may imply a high level of patient satisfaction with the outcome and continence effect of the KP. Despite the high prevalence of complication and revision, the pooled prevalence of pouch failure was 12.9%. Failure seems to be directly related to the length of follow-up as studies with follow-up durations < 10 years had a pooled rate of approximately 6%, compared with a rate of 14.4% in studies with longer follow-up periods. This finding may suggest that KP failures can be consequences of chronic issues that extend far past the time of pouch creation and may not necessarily be related to short-term complications. Given this observation, patients undergoing a KP may need sustained long-term follow-up with the diligence required by clinicians to ensure that patients are not lost to follow-up.

Failure did not significantly differ according to the geographic region of the studies. Interestingly, the meta-regression analysis did not find previous IPAA or the development of complications to be significantly associated with pouch failure. This finding implies that patients who had failed IPAA may still be eligible to have a KP and that, despite the expected high incidence of complications, the KP would be viable and functioning on long-term follow-up. A possible explanation of the high prevalence of complications and failure of KP is the small number of patients operated per year at each center, amounting to five patients per year on average. With such a small number of cases, it would be difficult to accrue sufficient experience and achieve a learning curve. An association between the hospital volume and outcomes of elective colorectal surgery including complications and reoperation has been previously reported [38].

The present systematic review represents a dedicated, focused analysis of the pooled prevalence, types, and risk factors for complications after KP creation. Subgroup analyses of the outcomes according to the year and region of study and follow-up duration added further information to the current literature. The results of this review were consistent with the experience of the senior author of this study, who has routinely treated patients with continent ileostomies for > 35 years and has created, revised, and excised numerous continent ileostomies.

The main limitations of this review include the retrospective nature and low quality of the studies reviewed. In addition, there was a high level of statistical heterogeneity within the outcomes. Reporting of complications was not consistent among the studies, which might be expected given the lack of standard of reporting morbidity after continent ileostomy. Nipple valve complications, albeit being the most common type of complications, were not exclusively reported in all studies. Moreover, during the period of our review, IPAA was the globally preferred operation for patients with MUC or FAP to avoid a Brooke ileostomy. Therefore, selection bias may be a large contributing factor to these results. Some of the baseline data, such as BMI, were not reported by most of the studies, limiting the power of the meta-regression analyses performed. The review did not account for differences in surgeons’ experience and volume, which may have an impact on the prevalence of complications. Furthermore, adverse selection may have been caused by patients with failed IPAA undergoing a KP. Lastly, some surgeons have advocated performing a KP for patients with Crohn’s disease [18, 28]. This selection bias may also have had a major effect on the outcomes including helping to explain the failure of the prevalence of revision and excision to decrease with time. 

## Conclusions

The KP is a highly complex operation, as shown by a pooled complication prevalence of 60%, and, thus, should be only performed by experienced surgeons. Despite the high prevalence of complications and need for revisional surgery, patients are keen to preserve their KP probably because they can improve their quality of life. Increased BMI and a previous failed IPAA are risk factors for pouch complications, but not failure. The incidence of KP failure is expected to increase with longer follow-up.

## Supplementary Information

Below is the link to the electronic supplementary material.

Supplementary file1 (DOCX 18 KB)

Supplementary Supplementary Fig. 1. Leave-one-out meta-analyses of the main outcomes file2 (JPG 219 KB)

Supplementary Supplementary Fig. 2. Funnel plot for assessment of publication bias file3 (JPG 126 KB)

## Data Availability

No datasets were generated or analyzed during the current study.
