# Supplementary material for: Complications and failure after Kock continent ileostomy: A systematic review and meta-analysis
Source: Tech Coloproctol. 2024 Oct 1;28(1):135. doi: 10.1007/s10151-024-03018-x (PMC11445325; doi:10.1007/s10151-024-03018-x)
Supplement: Supplementary file 1 — Supplementary file1 (DOCX 18 KB) [file 10151_2024_3018_MOESM1_ESM.docx]

**Appendix Table 1.** Results of quality assessment of non-randomized studies using ROBINS-1 tool

|  | **Bias due to confounding** | **Bias in selection of participants in the study** | **Bias in classification of intervention** | **Bias due to deviation from the intended intervention** | **Bias due to missing data** | **Bias in measurement of outcomes** | **Bias in selection of the reported result** | **Overall risk** |
| --- | --- | --- | --- | --- | --- | --- | --- | --- |
| **Ecker et al, 2022** | Some risk | Some risk | Low | Low | Low | Low | Low | Some risk |
| **Risto et al, 2022** | Some risk | Some risk | Low | Low | Low | Low | Low | Some risk |
| **Ecker et al, 2022** | Some risk | Some risk | Low | Low | Low | Low | Low | Some risk |
| **Risto et al, 2021** | Some risk | Some risk | Low | Low | Some risk | Low | Low | High |
| **Aytac etal, 2019** | Some risk | Some risk | Low | Low | Low | Low | Low | Some risk |
| **Aytac etal, 2017** | Some risk | Some risk | Low | Low | Low | Some risk | Low | High |
| **Sunde et al, 2017** | Some risk | Some risk | Low | Low | Low | Low | Low | Some risk |
| **Mukewar et al, 2014** | Some risk | Some risk | Low | Low | Low | Low | Low | Some risk |
| **Parc et al, 2011** | Some risk | Some risk | Low | Low | Low | Low | Low | Some risk |
| **Lian et al, 2009** | Some risk | Some risk | Low | Low | Low | Low | Low | Some risk |
| **Hoekstra et al, 2009** | Some risk | Some risk | Low | Low | Low | Low | Low | Some risk |
| **Wasmuth et al, 2009** | Some risk | Some risk | Low | Low | Some risk | Low | Low | High |
| **Denoya et al, 2008** | Some risk | Some risk | Low | Low | Low | Low | Low | Some risk |
| **Wasmuth et al, 2007** | Some risk | Some risk | Low | Low | Some risk | Low | Low | High |
| **Nessar et al, 2006** | Some risk | Some risk | Low | Low | Some risk | Low | Low | High |
| **Delaini et al, 2005** | Some risk | Some risk | Low | Low | Low | Low | Some risk | High |
| **Berndtsson et al, 2004** | Some risk | Some risk | Low | Low | Low | Low | Low | Some risk |
| **Castillo et al, 2005** | Some risk | Some risk | Low | Low | Low | Low | Low | Some risk |
| **Lepistö et al, 2005** | Some risk | Some risk | Low | Low | Some risk | Low | Low | High |

**Appendix Table 2.** Assessment of certainty of evidence using the GRADE approach

| **Certainty assessment** | | | | | | | **№ of events/patients** | **Certainty** | **Importance** |
| --- | --- | --- | --- | --- | --- | --- | --- | --- | --- |
| **№ of studies** | **Study design** | **Risk of bias** | **Inconsistency** | **Indirectness** | **Imprecision** | **Other considerations** |  |  |  |
| 16 | observational studies | Serious ^a^ | Very serious ^b^ | Not serious | Serious ^c^ | None | 859/1530 (56.1%) | ⨁◯◯◯ Very low | CRITICAL |
| 11 | observational studies | Serious ^a^ | Very serious ^b^ | Not serious | Not serious | None | 351/735 (47.8%) | ⨁◯◯◯ Very low | CRITICAL |
| 15 | observational studies | Serious ^a^ | Very serious ^b^ | Not serious | Not serious | None | 251/1887 (13.3%) | ⨁◯◯◯ Very low | CRITICAL |

**CI:** confidence interval

#### Explanations

a. Most studies had some risk of bias

b. The I^2^ index was >75%

c. The 95% Confidence Interval was wide
