# Supplementary figures and images for: Complications and failure after Kock continent ileostomy: A systematic review and meta-analysis
Source: Tech Coloproctol. 2024 Oct 1;28(1):135. doi: 10.1007/s10151-024-03018-x (PMC11445325; doi:10.1007/s10151-024-03018-x)

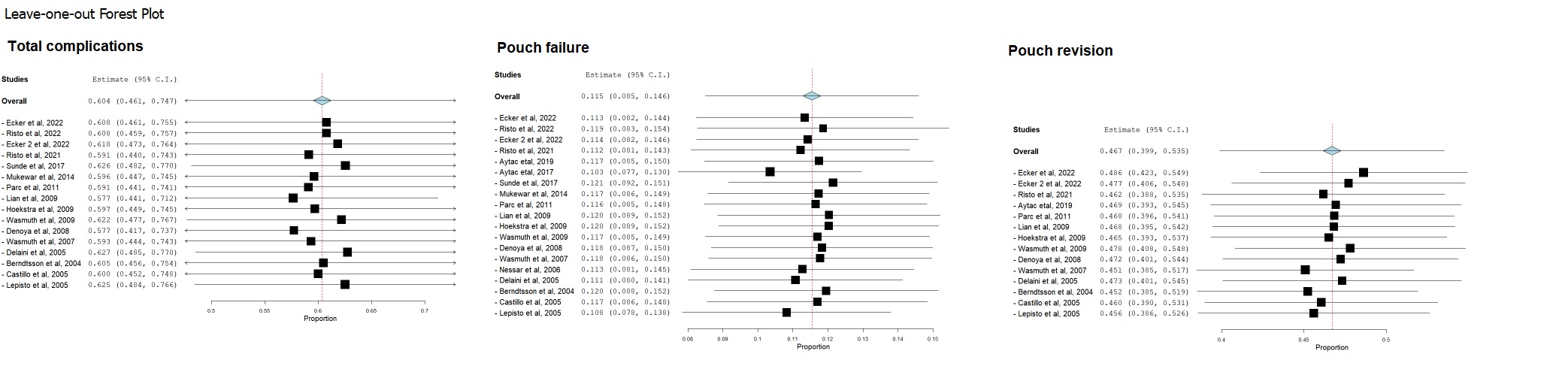

Supplement: Supplementary file 2 — Supplementary Supplementary Fig. 1. Leave-one-out meta-analyses of the main outcomes file2 (JPG 219 KB) [file 10151_2024_3018_MOESM2_ESM.jpg]

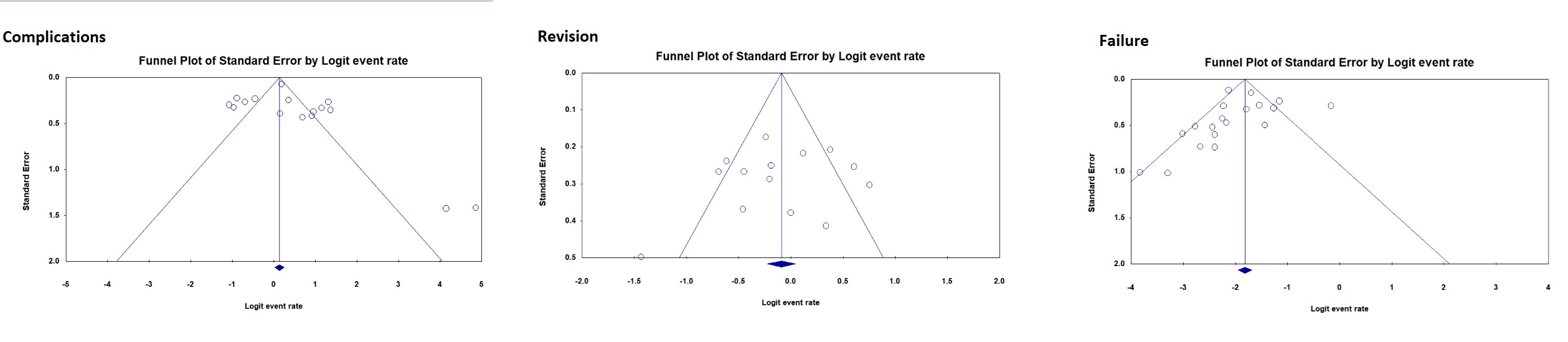

Supplement: Supplementary file 3 — Supplementary Supplementary Fig. 2. Funnel plot for assessment of publication bias file3 (JPG 126 KB) [file 10151_2024_3018_MOESM3_ESM.jpg]
